# AFAR Scientific Awards: Irving Wright Award, Terrie Fox Wetle Health Services Award, George Martin Mentoring Award

**DOI:** 10.1093/geroni/igaf122.1105

**Published:** 2025-12-31

**Authors:** Steven Austad

## Abstract

The Irving S. Wright Award of Distinction Lecture will feature an address by the 2025 recipient, David B. Allison, PhD. The Terrie Fox Wetle Award lecture will feature an address by the 2025 recipient, Lauren Hunt, PhD, RN, FNP. The George M. Martin Lifetime Achievement in Mentoring Award will also be presented at this event to 2025 recipient, Arlan Richardson, PhD. These awards are given by the American Federation for Aging Research, Inc.

